# Tools for the Assessment of the Malnutrition Status and Possible Interventions in Elderly with Cardiovascular Diseases

**DOI:** 10.3390/jcm10071508

**Published:** 2021-04-04

**Authors:** Elisabetta Tonet, Roberta Campana, Serena Caglioni, Federico Gibiino, Alessio Fiorio, Giorgio Chiaranda, Silvia Zagnoni, Gianni Casella, Gianluca Campo

**Affiliations:** 1 Cardiovascular Institute, Azienda Ospedaliero-Universitaria di Ferrara, 44124 Cona, Italy; robertacampana22@gmail.com (R.C.); s.caglioni@studenti.uniba.it (S.C.); federico.gibiino@hotmail.it (F.G.); alessiofiorio@virgilio.it (A.F.); cmpglc@unife.it (G.C.); 2 Department of Public Health, AUSL Piacenza, and Sport Medicine Service, 29121 Piacenza, Italy; g.chiaranda@ausl.pc.it; 3 Department of Cardiology, Ospedale Maggiore, 40133 Bologna, Italy; zagnonis@gmail.com (S.Z.); gianni.casella@ausl.bologna.it (G.C.)

**Keywords:** malnutrition, elderly, aortic stenosis, heart failure, coronary artery disease

## Abstract

Malnutrition represents a common and important feature in elderly people affected by cardiovascular diseases. Several studies have investigated its prevalence and prognostic role in most clinical settings, including cardiovascular disease. However, in daily practice it usually remains unrecognized and consequently untreated. The present review was ideated to answer the main questions about nutritional status assessment in patients with cardiovascular disease: why, when, where, how to evaluate it, and what to do to improve it. The three main cardiovascular diseases, namely aortic stenosis, ischaemic heart disease, and heart failure were considered. First, the main evidence supporting the prognostic role of malnutrition are summarized and analyzed. Second, the main tools for the assessment of malnutrition in the hospital and outpatient setting are reported for each condition. Finally, the possible strategies and interventions to address malnutrition are discussed.

## 1. Introduction

Over recent years, the mean age of patients with cardiovascular disease has been significantly increasing. Based on this, cardiologists should become confident with new risk factors characterizing the elderly. Nutritional status is one of them. Nutritional status is a marker of healthy status related to the risk of developing sarcopenia, cachexia, frailty, and disability. Frequently, disorders on nutritional status are associated with worsening of the physical performance and then with the overall condition of the patient. The universe of the nutritional status includes some definitions that must be clarified. Malnutrition is a chronic state characterized by over- and undernutrition and inflammatory status, which changes body composition [1]. Undernutrition is defined as an imbalance in the body’s energy intake and requirements. Overnutrition corresponds to an oversupplied intake of nutrients. Elderly patients are more often characterized by undernutrition because of age-related changes in taste, smell, and appetite, and also disease-related inflammation that can contribute to declines in appetite and changes in how the body processes nutrients [1,2].

The nutritional status can be easily investigated by the application of questionnaires, exams and scales. Cardiologists, and in general physicians, challenged with elderly with cardiovascular disease should be confident with malnutrition assessment for two main reasons. First, malnutrition deserves an important prognostic role. Patient’s nutritional status and its assessment should be considered part of the risk stratification in elderly. Second, malnutrition should be considered as an actionable risk factor. The presence of malnutrition could guide cardiologists in the implementation of corrective strategies potentially associated with a better outcome. These strategies include diet suggestions, nutritional supplements, and physical activity. They have to be individualized according to patient’s characteristics and comorbidities in order to plan the best secondary prevention program.

Therefore, the aim of the present review was summarizing evidence, prognostic role, and possible therapeutic strategies between nutritional status in elderly and the most common cardiovascular diseases in order to help cardiologists to perform nutritional evaluation first-hand.

## 2. Methods

A Medline search of full-text articles published in English until November 2020 was performed.

**Figure 1:**
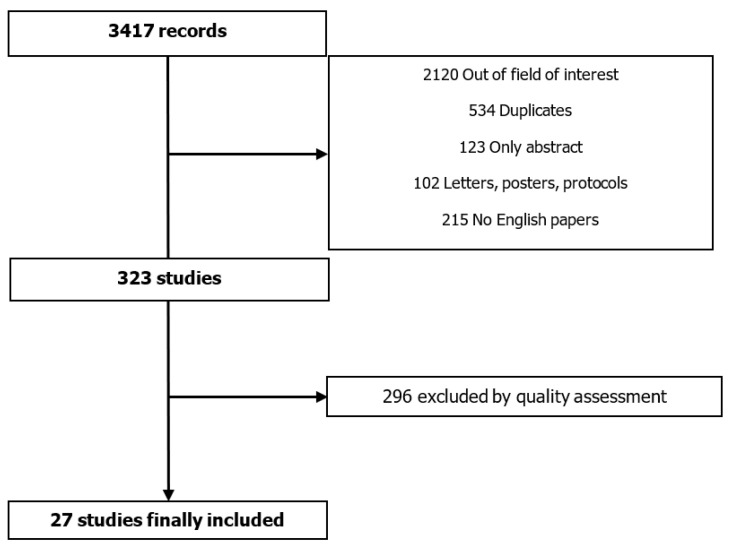
Methodological strategy.

Overall, 3417 records were identified. The search terms were: ((nutritional status) AND ((elderly) OR (older)) AND ((cardiovascular diseases) OR (aortic stenosis) OR (heart failure) OR ((myocardial infarction) OR (MI))). Only papers published in English and in peer-reviewed journals were selected. After evaluation of the title and the abstract, a total of 323 studies were analyzed as full text. The quality of the selected papers was tested using MINORS criteria [3]. Unblinded reviewers performed the analysis of the full texts for quality assessment. Discrepancies between reviewers were solved by consensus. The maximum score obtained was 14 and the minimum was 8. We included in the present review only studies obtaining a score of 10. A total of 27 papers were then considered for this overview (Figure 1).

## 3. Results

The following three main cardiovascular diseases were considered: aortic stenosis (AS), coronary artery disease (CAD), and heart failure (HF). The main characteristics of the most important tools used in these settings are summarized in Table 1.

### 3.1. Aortic Stenosis

#### 3.1.1. Why Assess Malnutrition

AS is the most common degenerative valve disease affecting elderly. This chronic, progressive disease is characterized by a prolonged inflammatory process that may contribute to the reduction of mobility and appetite and the loss of muscle mass. These typical features are of paramount importance, representing real risk factors for adverse outcomes in elderly [6]. Specific tools should be used in order to better estimate malnutrition; as a matter of fact, traditional markers of nutritional status, such as body mass index and body weight, demonstrated that they were not reliable indicators of malnutrition [6,7]. Data from the literature have reported a high rate of malnutrition in AS patients (about 65% in adults >65 years old) and its association with increased risk of mortality, hospital readmission, and longer length of hospital stay [7]. The predictive value of nutritional assessment would play a fundamental role in the evaluation elderly undergoing surgical aortic valve replacement (SAVR) and transcatheter aortic valve replacement (TAVR). In fact, it could help to choose the best strategy for each patient, and in patients considered for TAVR it could help acknowledge the possibility of futility. Indeed, Emami et al. [10] demonstrated that malnourished patients (defined according to Healthcare Cost and Utilization Project) undergoing TAVR experienced a higher rate of mortality (10.4% vs. 2.2%, *p* < 0.001); furthermore, the incidence of functional decline and poor outcomes at one year after was >50% in frail elderly. It has been demonstrated that one-year all-cause mortality occurred in the 27.7% of malnourished patients undergoing aortic valve replacement, in the 16.3% of patients at risk for malnutrition, and in the 9.7% of the well-nourished patients (*p* < 0.001) [6]. In addition, it has been shown that malnutrition attenuates the patient’s immune response, leading to delayed wound healing after surgery and consequent infective complications [7].

#### 3.1.2. When, How, and Where to Assess Malnutrition

In recent years, several studies have investigated and validated different tools to assess malnutrition in patients affected by aortic stenosis (Table 2 shows the main results) [4,5,6,7,8,9,9,10,11,12]. Nutritional assessment of AS should be performed during hospitalization for any reason with the evidence of symptomatic AS or during outpatient cardiological visits. Regardless of the score used, the prognostic value of malnutrition emerges to be of paramount importance. Considering the most important studies about malnutrition in AS, the most appropriate time to carry out a nutritional status evaluation could be:

- At the moment of the first diagnosis of symptomatic AS to obtain a comprehensive assessment of the patient and identify subjects presenting excessive risk for any type of intervention;
- When choosing the best type of replacement (TAVR or SAVR) for each patient in order to perform a better risk stratification;
- After aortic valve replacement, aiming at estimating residual risk.

The necessary elements to be considered to choose the best tool are the patients’ age, the co-presence of other unrecognized risk factors such as low physical performance and cognitive impairment, and the settings of patients’ assessment, as mentioned earlier. Therefore, for the successful and effective assessment of an AS patient’s nutritional status, the tool used should present four fundamental characteristics. It should be easy to perform, include a physical and cognitive function evaluation, have a strong predictive value, and it should not be based on laboratory values only. In view of this, when treating AS patients, the Mini Nutritional Assessment-Short Form (MNA-SF) and the Essential Frailty Tool (EFT) seem to be the best choices as they are easy to perform, not time-consuming, and with a well-established predictive value.

#### 3.1.3. Possible Interventions and Ongoing Studies

**Figure 2:**
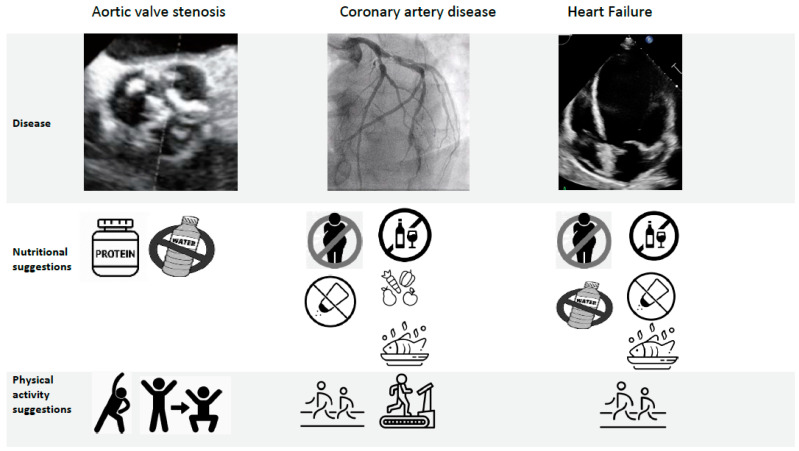
Nutritional and physical activity suggestions currently available in cardiovascular diseases Aortic stenosis: consider protein supplement before and early after surgical aortic valve replacement (SAVR) and transcatheter aortic valve replacement (TAVR), fluid management, ad hoc physical activity in order to improve lean mass. Coronary artery disease: energy intake should be limited to the amount of energy needed to maintain (or obtain) a healthy weight (BMI < 25 kg/m^2^); <5 g of salt per day; 30–45 g of fiber per day from wholegrain products, fruits, and vegetables; fish at least twice a week, one being oily fish; avoid excessive alcohol intake; aerobic physical activity. Heart failure: eat healthy and keep a healthy weight, avoid excessive salt intake (<6 g/day), abstain from or avoid excessive alcohol intake, fluid restriction, an ω-3 polyunsaturated fatty acids preparation may be considered, physical activity.

Ongoing research aims to investigate feasibility and benefits of nutritional status improvement in AS patients before replacement. Figure 2 shows some diet and lifestyle suggestions, which are just the evidence available up to now [1]. Currently, there are two ongoing trials considering cardiac prehabilitation before aortic valve replacement: the “Prehabilitation to Improve Functional and Clinical Outcomes in Patients With Aortic Stenosis” (TAVR-FRAILTY, NCT02597985) takes into consideration the functional exercise capacity (evaluated with a 6 min walking test) and the “PERFORM-TAVR” trial (PERFORM-TAVR, NCT03522454), which is evaluating the effects of a dietary supplement and a home-based supervised exercise program in patients suitable for TAVR.

### 3.2. Coronary Artery Disease 

#### 3.2.1. Why Assess Malnutrition

CAD is the leading cause of mortality and disability worldwide. The incidence of myocardial infarction (MI) is especially high in the elderly, and it is expected to increase because of the aging of the population [13]. Despite the improvement of strategies, technologies, drugs, and materials, the elderly remains the subgroup of MI patients with the worst prognosis. They show a higher rate of cardiovascular death after MI and 30-day hospital readmission for HF than their younger counterparts [13]. Furthermore, long-term follow-up data showed that two-year occurrence of cardiac death and MI is significantly more frequent in patients aged 75 years and over [14]. These data could be related to several comorbidities and unrecognized, but prognostically significant, risk factors, including malnutrition. Tonet et al. reported that 40% of patients >70 years old admitted for acute coronary syndrome were at risk for malnutrition [15]. Considering stable CAD, Wada et al. [16] reported that 49% and 24% of patients were at mild and moderate–severe risk of malnutrition, respectively. Table 3 shows the main studies in the setting of stable CAD [8,16,17,18] and acute MI [15,19,20,21,22], evaluating the prognostic value of malnutrition assessed by various tools.

#### 3.2.2. When, How, and Where to Assess Malnutrition

As previously reported, two clinical scenarios could require nutritional assessment for better risk stratification of CAD patients:

- Acute setting of MI;
- Outpatient visits of elderly with stable CAD.

During hospital admission, because of MI, the best tool should be easy and fast; MNA-SF and CONUT could represent the best choice, also considering the strong evidence of their prognostic value. For elderly with stable CAD, a more comprehensive tool could be used, such as the PNI or CONS; of note, these scores do not include cholesterol levels, which are influenced by chronic statin therapy in these patients.

#### 3.2.3. Possible Interventions

In relation to the strategies for nutritional status improvement, hitherto, there are no validated supplements or programs. As shown in Figure 2, suggestions provided by current guidelines are related to food intake regulation; these indications are useful both in young and older adults with CAD and are only partly aimed at improving lean mass and avoiding malnutrition and sarcopenia. The awareness that nutritional status has a strong prognostic value in elderly patients with CAD could guide secondary prevention programs addressing nutritional strategies’ implementation.

### 3.3. Heart Failure

#### 3.3.1. Why Assess Malnutrition

Malnutrition is common in patients with chronic HF, with a prevalence of about 45% [23]. Its association with high mortality is well established. Chronic HF often leads to loss of appetite, malabsorption, and a catabolic state, perpetuating a vicious cycle of malnutrition, cytokine activation, and autonomic dysfunction. The prevalence of malnutrition in chronic HF varies depending on the screening tool used, and it has been reported to be as high as 69% in some chronic HF populations [24,25]. Regardless of the score used, malnutrition is an independent predictor of worsening HF and mortality. Table 4 shows the main studies about nutritional assessment in HF patients [26,27,28,29,30,31,32,33,34,35,36]. Likewise, malnourished patients hospitalized for acute HF have a three times greater mortality risk than their counterparts with normal nutritional status [23]. During the acute phase of HF, the hepatic congestion and gut edema may cause early satiety and nausea, reducing the food assumption and adsorption. As a consequence, HF patients develop an increasingly poor nutritional status. The association between nutritional status and 30-day mortality among elderly patients with acute HF has also been proven in the emergency department [37]. These findings suggest that the risk of malnutrition should be screened in older patients with acute HF in every context in order to plan the best strategy for each patient.

#### 3.3.2. When, How, and Where to Assess Malnutrition

The assessment of nutritional status in HF patients should take place in three different scenarios:

- Outpatient visits, as completion of the evaluation of patients with chronic HF;
- Emergency department, in order to identify patients at higher risk for brief-term mortality who could benefit from more intensive care;
- During hospital stay for acute HF with the aim of improving management strategies.

With this background, the best nutritional tool should be easy to perform and comprehensive of laboratory and self-reported data in order to understand the lifestyle of each patients at home. Furthermore, the tool chosen should not consider parameters that could be influenced by ongoing medications, such as cholesterol levels. Considering these features, the best scores in this setting could be PNI, GNRI, and MNA, both in their long and short forms.

#### 3.3.3. Possible Interventions

The prognostic importance of malnutrition in HF patients has led to some strategies aimed at improving nutritional status (Figure 2). Dereli et al. [38] demonstrated that switching the anti-remodeling drugs to sacubitril/valsartan significantly improved the nutritional status in patients with HF with reduced ejection fraction. The PICINIC trial is a multicenter, randomized, controlled clinical trial that evaluated an individualized nutritional intervention in malnourished patients (according to MNA) hospitalized for acute HF. The primary endpoint was a composite of all-cause death or readmission for worsening HF, with a maximum follow-up of 12 months. The primary outcome occurred in 27.1% of patients in the intervention group and 60.7% of patients in the control group (HR 0.45; 95% CI 0.19–0.62, *p* = 0.0004). In total, 20.3% of patients died in the intervention group and 47.5% in the control group (HR 0.37, 95% CI, 0.19–0.72, *p* = 0.003). Readmission due to HF was also lower in the intervention group (10.2 vs. 36.1%, *p* = 0.001). Numbers needed to treat (NNTs) were 2.5 for the composite endpoint and 4 for all-cause mortality. These data show how a nutritional intervention in malnourished hospitalized patients with HF reduces the risk of all-cause death and the risk of readmission for worsening HF [39].

## 4. Discussion

### Beyond the Malnutrition Condition

An important concept emerged from previous studies on the elderly: new risk factors involved in the complexity of these patients include not only malnutrition, but also low physical performance and cognitive decline [40]. While the latter is not frequent in a cardiological setting, malnutrition and low physical performance are often encountered in patients with cardiovascular diseases [41]. These points could be considered two sides of the same coin. As a matter of fact, they are strictly related: on the one hand, poor nutritional status determines a loss of muscle mass with a consequent poor physical performance. On the other hand, low physical performance slows the metabolism and reduces appetite, resulting in lower intake and absorption of nutrients. With this background in mind, nutritional status assessment and improvement are important, but they cannot be considered separated from a physical activity intervention. As previously reported, their impact on prognosis of these patients is of paramount importance. As a matter of fact, the trajectory of elderly patients with cardiovascular diseases and low physical performance and/or malnutrition has been demonstrated to go rapidly down [41]. Additionally, it has to be considered that nutritional status and physical performance reflect the global health status, so that management of these patients should be regulated, also taking into account these characteristics. For example, in the setting of CAD, nutritional status assessment and low physical performance evaluation could guide the invasive strategy, recognizing those patients in which benefits of revascularization could be weakened by pre-frailty and frailty burden [41]. Therefore, the recognition of these risk factors has become fundamental in order to improve management and prognosis of elderly patients. A comprehensive assessment of elderly people with cardiac disorders should include both malnutrition and physical performance evaluation. The information should guide tailored intervention of secondary prevention, including diet, nutritional supplements [42], and exercise programs (supervised in well-established facilities or home-based) [43].

## 5. Conclusions and Future Directions

Data incontrovertibly show that the nutritional status is related to poor prognosis in elderly patients affected by cardiovascular diseases, and it is relevant both in the acute and chronic setting. There are several validated context-specific tools to evaluate these patients. The assessment of nutritional status and physical performance should be integrated in the routine clinical practice because it could help choose the best diagnostic and therapeutic pathway for each patient. If, on the one hand, the importance of nutritional status assessment has been widely demonstrated, on the other, there are not enough data showing the benefits of nutritional intervention. How to implement nutritional status of these patients and whether its improvement has a prognostic relevance is still unknown and future studies are clearly on demand. 

## Figures and Tables

**Table 1:** Main scores for the assessment of nutritional status.

Score	Brief Description	Cut off Values	Setting of Validation
CONUT [4] Controlling Nutritional Index	Assess the risk of malnutrition giving points for each parameter, then summarize the point: Albumin, g/dL Total cholesterol, mmol/L Lymphocyte count, ×10^9^/L	0–1 = no risk 2–4 = mild risk 5–8 = moderate risk 9–12 = severe risk	Aortic stenosis Coronary artery disease Heart failure
GNRI [5] Geriatric Nutritional Risk Index	Simple screening tool to assess nutritional status based on serum albumin and body mass index. GNRI = 14.89× serum albumin [g/dL] + 41.7× [actual body weight/ideal body weight]	>104 no risk 98–104 moderate risk <98 severe risk	Aortic stenosis Coronary artery disease Heart failure
PNI [5] Prognostic Nutritional Index	Assess the risk of malnutrition with the formula: 10× serum albumin (g/dL) + 0.005× total lymphocyte count (mm^3^)	>38 no risk 35–38 moderate risk <35 severe risk	Aortic stenosis Coronary artery disease Heart failure
MNA-SF [6] Mini Nutritional Assessment -Short Form	Able to identify multifactorial causes of nutritional risk specifically in elderly.	>24 scores = no risk 17–23.5 scores = at risk of malnutrition <17 scores = malnourished	Aortic stenosis Coronary artery disease Heart failure
	A questionnaire consisting of 18 components grouped into four components, which are anthropometry data, general status, dietary habits, self-perceived health, and nutrition status		
EFT [7] Essential Frailty Toolset	The EFT is scored 0 (least frail) to 5 (most frail) based on the following four items: preprocedural anemia; hypoalbuminemia; lower-extremity muscle weakness, defined as a time of >15 s or inability to complete five sit-to-stand repetitions without using arms; and cognitive impairment, defined as a score of <24 on the Mini-Mental State	0–2 low risk of frailty 3–5 high risk of frailty	Aortic stenosis
Combined Objective Nutritional Score [8]	Assessment the risk of malnutrition giving 1 point each for: high CONUT score (3–12), low GNRI (<98) or low PNI (<45)	0 = no risk 1–2 = moderate risk 3 = severe risk	Coronary artery disease
SGA [9] Subjective Global Assessment	A validated tool consisting of clinical history (weight loss history, dietary intake changes, gastrointestinal symptoms persisting for more than 2 weeks, and functional capacity) and physical examination (subcutaneous fat, muscle wasting, ankle and sacral edema, and ascites	Based on nutrition rating: SGA A = well-nourished SGA B = moderate or suspected undernourished SGA C = severely undernourished	Aortic stenosis

**Table 2:** Main studies about nutritional tools in aortic stenosis.

Most Important Studies	Number of Patients	Nutritional Tool Used	Mean Age (Years)	Setting	Results
Goldfarb et al. [6]	1158	MNA-SF	81.3 ± 6.1	Hospital	Pts with malnutrition have 3-fold increase in mortality one year following AVR
Afilalo et al. [7]	1020	EFT	82 (77–86)	Hospital	Pts with EFT > 4 have: - 3-fold increase in 30- day mortality - Good maker to evaluate futility
Honda Y. et al. [4]	150	CONUT	86 ± 5	Hospital	Association between CONUT score and increase mortality after TAVR.
Hebeler K. et al. [11]	470	Serum albumin level	81.7 ± 7.9	Hospital	Albumin is predictive of 1-year mortality and may be a useful variable to include in TAVR risk scores.
Lee K et al. [5]	412	GNRI CONUT	78.7 ± 5.2	Hospital	GNRI and CONUT score reflected mortality risk. Lower GNRI (≤ 98) was the only independent predictor of all-cause death at 1 year
Okuno T et al. [12]	95	GNRI CONUT PNI	84 (81–88)	Hospital	CONUT score and PNI were associated with 1-year clinical outcomes especially with 1-year all-cause mortality in patients undergoing TAVR. CONUT score and PNI might have better predictive values than GNRI
Wernio et al. [9]	101	f-MNA, 7-SGA, low concentrations of total cholesterol, LDL-cholesterol, and prealbumin were considered	74.6 ± 5.2	Hospital	In malnourished patients the risk of postsurgery complications increased 1.22 times. Unintentional weight loss of >2.8% in the six months preceding surgery predicted death within the first year after AVR surgery

Legend: AVR = aortic valve replacement; CONUT = Controlling Nutritional Status; EFT = Essential Frailty Tool; f-MNA = Low Mini Nutritional Assessment; GNRI = Geriatric Nutritional Risk Index; MNA-SF = Mini Nutritional Assessment Short Form; SAVR = Surgical Aortic Valve Replacement; TAVR = Transcatheter Aortic Valve Replacement; 7-SGA = Subjective Global Assessment.

**Table 3:** Main studies about nutritional tools in chronic and acute ischemic heart disease.

	Most Important Studies	Number of Patients	Nutritional Tool Used	Mean Age (years)	Setting	Results
Chronic ischemic heart disease	Wada et al. [16]	2853	GNRI	69 ± 10	Hospital	Lower GNRI was an independent predictor of all-cause mortality (HR 1.55, CI 1.08–1.90, *p* < 0.0001) and cardiac death (HR 144, CI 1.08–1.90, *p* < 0.01)
	Wada et al. [17]	1984	CONS	68.2 ± 9.6	Hospital	CONS of 3 showed 2.91-fold (95% (CI) 2.10–4.00; *p* < 0.0001) and 2.16-fold (95% CI 1.15–3.92; *p* = 0.02) increases in risk of mortality and cardiac mortality compared with patients with a CONS of 0.
	Wada et al. [8]	1988	PNI	69.7 ± 9.4	Hospital	Lower PNI scores are correlated with the increased cumulative incidence of MACE and all-cause death (*p* < 0.0001 each). PNI is independently associated with cardiovascular outcomes after adjusting for these risk factors.
	Kunimura et al. [18]	1004	CONUT score combined with BMI	73 ± 9	Hospital	High CONUT score + normal BMI showed a 2.72-fold increase in the incidence of MACE (95% CI 1.46–5.08, *p* = 0.002)
Acute ischemic heart disease	Oduncu V et al. [19]	1706	Serum albumin	61.3 ± 12.3	Hospital	Hypoalbuminemia at admission is a strong independent predictor for long-term mortality and development of advanced HF in patients with STEMI undergoing *p*-PCI.
	Tonet E et al. [15]	908	MNA-SF	82 ± 6	Hospital	MNA-SF is an independent predictor of all-cause mortality (HR 0.76, 95% CI 0.68–0.84 for single change unit). The MNA-SF score improved the GRACE score’s ability to discriminate subjects at risk of death.
	Keskin M et al. [20]	1823	PNI	58 ± 11	Hospital	Long-term mortality was also significantly higher in the group with PNI < 44, confirmed even after adjustment for possible confounders
	Komici K et al. [21]	174	MNA	74.2 ± 7	Hospital	MNA showed a significant and independent impact on mortality (HR = 0.56, 95% CI = 0.42–0.73)
	Basta G et al. [22]	945	CONUT, PNI	78 ± 9	Hospital	CONUT > 2 but not PNI < 35, has the highest event rate for all-cause death (*p* < 0.001). CONUT but not the PNI was associated with increased risk of all-cause death for an unadjusted model.

Legend: BMI = body mass index; CONS = combined objective nutritional score. For other abbreviations see Table 2.

**Table 4:** Main studies about nutritional tools in heart failure.

Most Important Studies	Number of Patients	Nutritional Tool Used	Mean Age (Years)	Setting	Results
Candeloro M et al. [26]	344	PNI	84 (65–101)	Hospital	PNI values ≤ 34 is associated with a twofold higher risk of overall mortality (HR 2.54; 95% CI, 1.52 to 4.24) and threefold higher risk of in-hospital mortality (HR 3.37; 95% CI, 1.14 to 9.95).
Iwakami et al. [27]	635	CONUT	78 ± 10	Hospital	CONUT score is independently associated with death (HR 1.26, 95% CI 1.11–1.42, *p* < 0.001).
Nishi I et al. [28]	482	CONUT	71.7 ± 13.6	Hospital	Demonstrate the usefulness of CONUT scores as predictors of short-term prognosis in hospitalized HF patients
Kato T et al. [29]	2466	CONUT	79 (70–85)	Hospital	The excess risk of high relative to low CONUT score for mortality and infection is significant (OR: 1.61, 95% CI: 1.05–2.44 and OR: 1.66, 95% CI: 1.30–2.12, respectively). The effect was incremental according to the score.
Sze S et al. [30]	265	PNI	76 (69–82)	Outpatient visit	A model, including CFS and PNI, increased c-statistic for mortality prediction from 0.68 to 0.84. Worsening frailty and malnutrition indices are strongly related to worse outcomes in patients hospitalised with HF.
Cheng YL et al. [31]	1673	PNI	75.8 ± 13.2	Hospital	PNI is independently associated with long-term survival in patients hospitalized for acute heart failure with either reduced or preserved left ventricular ejection fraction.
Nishi I et al. [32]	110	GNRI	78.5 ± 7.2	Hospital	GNRI at discharge is helpful to predict the long-term prognosis of elderly HFpEF patients
Sargento L et al. [33]	50	MNA	74.3 ± 6.3	Outpatient visit	Patients with malnutrition by the MNA-SF were at greater risk of death (HR = 8.0 *p* = 0.059) and hospitalization (HR 8.1 *p* = 0.008)
Sze et al. [34]	3386	CONUT, GNRI, PNI	75 (67–81)	Outpatient visit	Malnutrition is frequent in HF and it is strongly related to worse outcomes. Amongst the malnutrition scores, GNRI had the greatest incremental value.
Alatas O et al. [35]	628	GNRI, PNI, CONUT,	74.7 ± 11.8	Hospital	Though all objective nutritional indexes were associated with prognosis in elderly patients with acute heart failure, GNRI was superior to other scores in predicting in-hospital mortality.
Sze S et al. [36]	467	CONUT, GNRI, PNI MUST, MNA-SF	76 (69–82)	Outpatient visit	Among the 6 malnutrition tools studied, MNA-SF has the best classification performance in identifying significant malnutrition as defined by the combined index

Legend: HF = heart failure; HFrEF = heart failure with reduced ejection fraction; HFpEF = heart failure with preserved ejection fraction. For other abbreviations see Table 2and Table 3.

## Data Availability

Not applicable.
